# What is the impact of snakebite envenoming on domestic animals? A nation-wide community-based study in Nepal and Cameroon

**DOI:** 10.1016/j.toxcx.2021.100068

**Published:** 2021-06-05

**Authors:** Isabelle Bolon, Sara Babo Martins, Carlos Ochoa, Gabriel Alcoba, María Herrera, Henri Magloire Bofia Boyogueno, Barun Kumar Sharma, Manish Subedi, Bhupendra Shah, Franck Wanda, Sanjib Kumar Sharma, Armand Seraphin Nkwescheu, Nicolas Ray, François Chappuis, Rafael Ruiz de Castañeda

**Affiliations:** a Institute of Global Health, Department of Community Health and Medicine, Faculty of Medicine, University of Geneva, Chemin des mines 9, 1202, Geneva, Switzerland; b Institute for Environmental Sciences, University of Geneva, 66 boulevard Carl-Vogt, 1205, Geneva, Switzerland; c Division of Tropical and Humanitarian Medicine, Geneva University Hospitals (HUG), Department of Community Health and Medicine, Faculty of Medicine, 24 rue Micheli-du-Crest, Geneva 14, 1211, Switzerland; d Médecins Sans Frontières (MSF), Rue de Lausanne 78, 1202, Geneva, Switzerland; e Instituto Clodomiro Picado, Facultad de Microbiología, Universidad de Costa Rica, San José, 11501-2060, Costa Rica; f Ministère de l’Elevage, des Pêches et des Industries Animales (MINEPIA), Direction des Services Vétérinaires, Yaoundé, Cameroon; g Ministry of Agriculture and Livestock Development, Singhadurbar, Kathmandu, Nepal; h B.P. Koirala Institute of Health Sciences (BPKIHS), Buddha Road, Dharan, 56700, Nepal; i Centre International de Recherche, d’Enseignement et de Soins en Milieu Tropical (CIRES), BP 11 Akonolinga, Cameroon; j Cameroon Society of Epidemiology (CaSE), P.O.Box 1411, Yaoundé, Cameroon; k Faculty of Medicine and Biomedical Science, University of Yaoundé 1, Yaoundé, Cameroon

**Keywords:** Snakebite, Livestock, Community-based survey, Ethno-veterinary medicine, Antivenom, One Health

## Abstract

Snakebite envenoming is a life-threatening disease in humans and animals and a major public health issue in rural communities of South-East Asia and sub-Saharan Africa. Yet the impact of snakebite on domestic animals has been poorly studied. This study aimed to describe the context, clinical features, treatment, and outcomes of snakebite envenoming in domestic animals in Nepal and Cameroon. Primary data on snakebite in animals were recorded from a community-based nation-wide survey on human and animal snakebite in Nepal and Cameroon (*Snake-byte* project). Mobile teams collected data on snakebite in humans and animals in 13,879 and 10,798 households in Nepal and Cameroon respectively from December 2018 to June 2019. This study included 405 snakebite cases (73 in Nepal and 332 in Cameroon) in multiple types of animals. An interview with a structured questionnaire collected specific information about the animal victims.

Snake bites in animals took place predominantly inside and around the house or farm in Nepal (92%) and Cameroon (71%). Other frequent locations in Cameroon were field or pasture (12%). A large diversity of clinical features was reported in all types of envenomed animals. They showed either a few clinical signs (e.g., local swelling, bleeding) or a combination of multiple clinical signs. Only 9% of animal victims, mainly cattle and buffaloes and less frequently goats, sheep, and dogs, received treatment, predominantly with traditional medicine. The overall mortality of snakebite was 85% in Nepal and 87% in Cameroon.

Results from this nationwide study show an important impact of snakebite on animal health in Nepal and Cameroon. There is a need for cost-effective prevention control strategies and affordable snakebite therapies in the veterinary field to save animal lives and farmer livelihood in the poorest countries of the world. The WHO global strategy to prevent and control snakebite envenoming supports a One Health approach, which may help develop integrated solutions to the snakebite problem taking into account human and animal health.

## Highlights

• Analysis of 405 snakebite cases in animals collected through a nationwide community-based survey in Nepal and Cameroon.
• Snakebite in domestic animals occurred predominantly inside and around the house or farm.
• Clinical features of envenoming were diverse in all types of animals.
• Only 9% of animal victims were treated, mainly cattle and buffaloes, predominantly with traditional medicine.
• The overall mortality of snakebite in animals was 85% in Nepal and 87% in Cameroon.

## 1 Introduction

Snakebite envenoming (snakebite) is an acute, potentially life-threatening disease that affects both people and domestic animals. Snakebite is a major human public health issue in rural communities of South-East Asia and sub-Saharan Africa (Gutiérrez et al., 2017), yet the understanding of the impact of this disease on domestic animals is very limited. Veterinary literature is biased geographically and in terms of the type of animals (Bolon et al., 2019). Most studies focus on pets (i.e., dogs, cats) and animals for leisure (i.e., horses) in the USA (Fielding et al., 2011; Gilliam and Brunker, 2011; Witsil et al., 2015) and Australia (Cullimore et al., 2013; Padula et al., 2016; Bamford et al., 2018), and to a lesser extent in Europe (Anlén, 2008; Lervik et al., 2010; Sutton et al., 2011; Turkovic et al., 2015), Middle-East (Segev et al., 2004), Latin America (Koscinczuk et al., 2000), and South Africa (Leisewitz et al., 2004). Although 70% of the world's poor raise livestock (FAO, 2009), the impact of snakebite on cattle, sheep, goats, pigs, etc. has been largely neglected in snakebite endemic countries (Bolon et al., 2019). An exception for this is Costa Rica, where snakebite and other animal attacks cause about 11,000 cattle losses per year (National Institute of Statistics and Census, 2019). Other Latin American countries have reported snakebite in livestock including cattle and horses in Colombia (Gómez et al., 2014) and cattle and sheep in Brazil (Mendez and Riet-Correa, 1995; Tokarnia and Peixoto, 2006; do Rego Leal et al., 2013). In India, a retrospective study done over 14 years, described the clinical presentation of 98 cattle envenomed by viper snakes (Bhikane et al., 2020). Swelling, bleeding at the site of the bite, and lameness were the main clinical signs. The survival rate was 88% in these animals that were all treated with polyvalent antivenom at a veterinary hospital (Bhikane et al., 2020). Apart from this larger study, only a few clinical case reports exist for snakebite in cattle in India and Pakistan (Farooq et al., 2014; Kachhawa et al., 2016; Senthilkumar et al., 2018; Ali and Arul, 2020; Arul et al., 2020), in goats and sheep in India (Sivaraman et al., 2016; Prasanth et al., 2017; Sakhare et al., 2019; Venkatesakumar et al., 2020), in pigs in Zimbabwe (Stewart, 1974), and poultry in Nigeria (Onoviran et al., 1976; Lawal et al., 1992). Chapman et al. (1998) also stressed the difficulty of gathering epidemiological and clinical data on snakebite in livestock during a hospital-based survey done in the Terai region in Nepal. The number of veterinarians and other animal health care practitioners, as well as healthcare services and epidemiological surveillance, are, in general, very limited in low and middle-income countries (LMICs) where snakebite is endemic, particularly in remote rural areas (Sen and Chander, 2003; Van den Bossche et al., 2004). Snakebite is not a notifiable disease, therefore is not officially reported in humans nor animals, and the burden of this disease is largely underestimated.

In line with the One Health framework proposed in Babo Martins et al. (2019) and the current WHO road map on snakebite (WHO 2019), the objective of this study was to describe the impact of snakebite in domestic animals. We analyzed the context, clinical features, treatment, and outcomes of snakebite in animals using primary data collected through the first community-based, nation-wide survey on human and animal snakebite in Nepal and Cameroon.

## 2 Methods

### 2.1 Data collection

Animal cases of snakebite were retrospectively recorded through a nation-wide, community-based, One Health, epidemiological survey on snakebite in Nepal and Cameroon (the “*Snake-byte*” project). The study design is described in detail in Alcoba et al. (2021). In brief, the *Snake-byte* project included a cross-sectional, multi-cluster, random household survey that collected data on snakebite in humans and domestic animals in 13,879 households in Nepal (Terai region) and 10,798 households in Cameroon, from December 2018 to June 2019. The *Snake-byte* project aimed primarily at assessing the impact of snakebite on human and animal health including snakebite incidence, morbidity and mortality. These data together with geo-spatial analysis will feed predictive models to map local and sub-national snakebite hotspots and accessibility to life-saving healthcare (e.g., antivenoms) to guide local and national public health policies for improved prevention and control of the disease. This paper focuses on the clinical veterinary aspects of the project, including signs of envenoming in animals, health outcomes, and treatment. The epidemiological data (i.e. incidence, geography, etc.) are published in other articles.

The household survey was conducted by mobile teams guided by a local community health worker, who could translate questions into the local language whenever needed. Data were recorded on electronic tablets using the KoBoCollect tool (Pham et al., 2019). Representatives of all selected households were asked about the occurrence of snakebite in the past year and human and domestic animal victims were included only if the bite occurred during that time. Domestic animals (animals hereafter) refer to livestock animals such as cattle, goats, sheep, pigs, poultry, etc., and work animals such as horses, donkeys, guard and shepherd dogs, etc., depending on each socio-ecological context in the study areas.

### 2.2 Questionnaire survey

Where snakebite was reported for animals, the animal owner (owner hereafter) further underwent an interview with a structured questionnaire to collect specific information on the animal victim. The questionnaire included questions on the circumstances of the snake bite (allowing for case classification), the scene of the bite, the part of the body that was bitten, the clinical signs, the resulting actions taken in terms of health care (e.g., seeking veterinary or traditional care, type of treatment administered), and the outcome of the snakebite (e.g., recovery, sequelae, death) (see survey questionnaire in supplementary materials). The questionnaire included close-ended questions providing a set of pre-defined responses. The respondent was allowed to propose further details, which were recorded as free text. The pre-defined responses for clinical signs (e.g., swelling, bleeding, hypersalivation, paralysis, etc.) (see supplementary materials) were based on our previous literature review on the impact of snakebite in animals (Bolon et al., 2019). When more than one animal of the same type was bitten on the same date in a given household (i.e. same episode), only one questionnaire was completed and the episode was counted as one snakebite case. The owner was then asked to answer the survey in reference to the most common answer in that group of animals. The questionnaire was available in English for Nepal and in French and English for Cameroon.

### 2.3 Ethics statement

Ethics approval was obtained from the *Comité National d’Ethique de la Recherche pour la Santé Humaine* in Cameroon (CNERSH, No. 2018/09/1208), the National Health Research Council in Nepal (NHRC Reg.no. 585/2018), and the *Commission Cantonale d’Ethique de la Recherche scientifique* in Geneva (CCER and Swiss Ethics Registry No. 2018-01331).

### 2.4 Data analysis

The raw data including 727 snakebite cases in animals were exported from Kobo-toolbox™ (Harvard Humanitarian Initiative) to Excel spreadsheets (Microsoft Corporation, USA) and then managed and curated in R (R-Core-Team., 2020). Cases were classified according to the description of the event provided by the respondent during the survey. The snakebite was considered *confirmed* if the event was witnessed, and clinical signs were consistent with snakebite envenomation (e.g., swelling, bleeding, ptosis, hypersalivation, respiratory difficulties, paralysis, or sudden death). The snakebite was considered *probable* if the event was not witnessed but there was evidence of contact with a snake (animal playing, fighting a snake, a snake found close to the animal) or fang marks were found, and clinical signs were consistent with snakebite envenomation. The snakebite was considered *suspected* if the event was not witnessed, there was no evidence of contact with a snake and no report of fang marks, but the animal victim was found alive or dead with clinical or post-mortem signs consistent with snakebite envenomation. Only confirmed and probable snakebites were included in the analysis. Suspected snakebite cases were not included because the diagnosis of snakebite was deemed too uncertain.

The analysis was descriptive and the frequency (%) of snakebite for the variables studied (e.g., location of the snake bite, body part bitten, treatment and outcome) was calculated for each type of animal bitten for Nepal and Cameroon. Cases of snakebite in bovids, including cattle and buffaloes, and equids, including horses and donkeys, were not disaggregated during the survey, and results are jointly presented for these groups in the results. Data on goats and sheep were collected separately but finally combined in the analysis since these animals are all small ruminants. Odds Ratio (OR) was used to measure the association between treatment and outcome with OpenEpi (Dean et al., 2013). Upset plots were produced with the UpSetR Shiny App (Lex et al., 2014).

## 3 Results

### 3.1 Scene of the snake bite

In total, there were 405 confirmed and probable snakebite cases including 73 cases in Nepal (43 poultry, 15 cattle or buffaloes, 14 goats and sheep, and one dog) and 332 cases in Cameroon (207 poultry, 50 goats and sheep, 35 cattle or buffaloes, 19 dogs, 11 pigs, 8 cats, and 2 horses or donkeys).

The bite to the animals occurred in various locations, including the owner's house or farm, the animal shed, or the field or pasture. Yet, for all types of animals combined, the great majority of snake bites took place inside and around the house or farm in Nepal (92%) and Cameroon (71%) (Table 1). Cases observed in field or pasture represented 12% of cases in Cameroon and only 4% in Nepal. In Cameroon, the reported locations per type of animals were more diverse than in Nepal with field/pasture being predominant in cattle and buffaloes (40%) and as frequent as around the farm in goats and sheep (34%).

**Table 1:** Distribution of snakebite cases in animals in Nepal and Cameroon by location of the snake bite event.

Scene of the bite	Types of animals in Nepal					Types of animals in Cameroon							
	Cattle & Buffalo (n = 15) n(%)	Goat & Sheep (n = 14) n(%)	Poultry (n = 43) n(%)	Dog (n = 1) n(%)	Total (n = 73) n(%)	Cattle & Buffalo (n = 35) n(%)	Horse & Donkey (n = 2) n(%)	Goat & Sheep (n = 50) n(%)	Pig (n = 11) n(%)	Poultry (n = 207) n(%)	Dog (n = 19) n(%)	Cat (n = 8) n(%)	Total (n = 332) n(%)
In the house/farm	10 (67)	6 (43)	33 (77)	0 (0)	49 (67)	4 (11)	0 (0)	1 (2)	0 (0)	111 (54)	0 (0)	3 (38)	119 (36)
Around the house/farm	2 (13)	6 (43)	9 (21)	1 (100)	18 (25)	9 (26)	1 (50)	17 (34)	6 (55)	75 (36)	4 (21)	4 (50)	116 (35)
In a pen/stall/coop/stable	0 (0)	1 (7)	0 (0)	0 (0)	1 (1)	4 (11)	0 (0)	2 (4)	3 (27)	13 (6)	0 (0)	0 (0)	22 (7)
In the field/pasture	1 (7)	1 (7)	1 (2)	0 (0)	3 (4)	14 (40)	0 (0)	17 (34)	1 (9)	4 (2)	3 (16)	0 (0)	39 (12)
In a plantation	0 (0)	0 (0)	0 (0)	0 (0)	0 (0)	0 (0)	0 (0)	2 (4)	0 (0)	0 (0)	3 (16)	0 (0)	5 (2)
Close to a river	1 (7)	0 (0)	0 (0)	0 (0)	1 (1)	0 (0)	1 (50)	0 (0)	0 (0)	0 (0)	7 (37)	0 (0)	8 (2)
In a forest	0 (0)	0 (0)	0 (0)	0 (0)	0 (0)	2 (6)	0 (0)	0 (0)	0 (0)	1 (0)	0 (0)	0 (0)	3 (1)
Unknown	1 (7)	0 (0)	0 (0)	0 (0)	1 (1)	2 (6)	0 (0)	11 (22)	1 (9)	3 (1)	2 (11)	1 (13)	20 (6)

### 3.2 Clinical features of snakebite in animals

#### 3.2.1 Part of the animal body bitten by the snake

Animal victims were bitten by snakes in diverse parts of their body including a limb, head-neck area, abdomen, or thorax. Large animals (e.g., cattle, buffaloes, horses, and donkeys) were mostly bitten on a limb (Table 2). Besides, the limb and head-neck area, abdomen and thorax were common bite sites in goats, sheep, pigs, and poultry. In poultry, the site of the bite was also frequently unknown. Dogs were predominantly bitten on the head-neck area (47%) or a limb (32%), while for cats this was the opposite (50% of bites reported on a limb and 13% on the head-neck area). Animals were only occasionally bitten on the udder or tail.

**Table 2:** Distribution of snakebite cases in animals in Nepal and Cameroon by part of the animal body bitten by the snake.

Body part bitten	Types of animals in Nepal					Types of animals in Cameroon							
	Cattle & Buffalo (n = 15) n(%)	Goat & Sheep (n = 14) n(%)	Poultry (n = 43) n(%)	Dog (n = 1) n(%)	Total (n = 73) n(%)	Cattle & Buffalo (n = 35) n(%)	Horse & Donkey (n = 2) n(%)	Goat & Sheep (n = 50) n(%)	Pig (n = 11) n(%)	Poultry (n = 199) n(%)	Dog (n = 19) n(%)	Cat (n = 8) n(%)	Total (n = 324) n(%)
Limbs	7 (47)	4 (29)	3 (7)	0 (0)	14 (19)	28 (80)	2 (100)	27 (54)	6 (55)	60 (30)	6 (32)	4 (50)	133 (41)
Head, face, or neck	4 (27)	3 (21)	11 (26)	0 (0)	18 (25)	3 (9)	0 (0)	10 (20)	1 (9)	34 (17)	9 (47)	1 (13)	58 (18)
Abdomen or thorax	1 (7)	7 (50)	1 (2)	1 (100)	10 (14)	2 (6)	0 (0)	6 (12)	2 (18)	57 (29)	2 (11)	0 (0)	69 (21)
Udder	1 (7)	0 (0)	0 (0)	0 (0)	1 (1)	0 (0)	0 (0)	0 (0)	0 (0)	0 (0)	0 (0)	0 (0)	0 (0)
Tail	0 (0)	0 (0)	0 (0)	0 (0)	0 (0)	1 (3)	0 (0)	2 (4)	0 (0)	7 (4)	1 (5)	0 (0)	11 (3)
Unknown	2 (13)	0 (0)	28 (65)	0 (0)	30 (41)	1 (3)	0 (0)	5 (10)	2 (18)	41 (21)	1 (5)	3 (38)	53 (16)

#### 3.2.2 Clinical signs of snakebite in animals

**Fig. 1:**
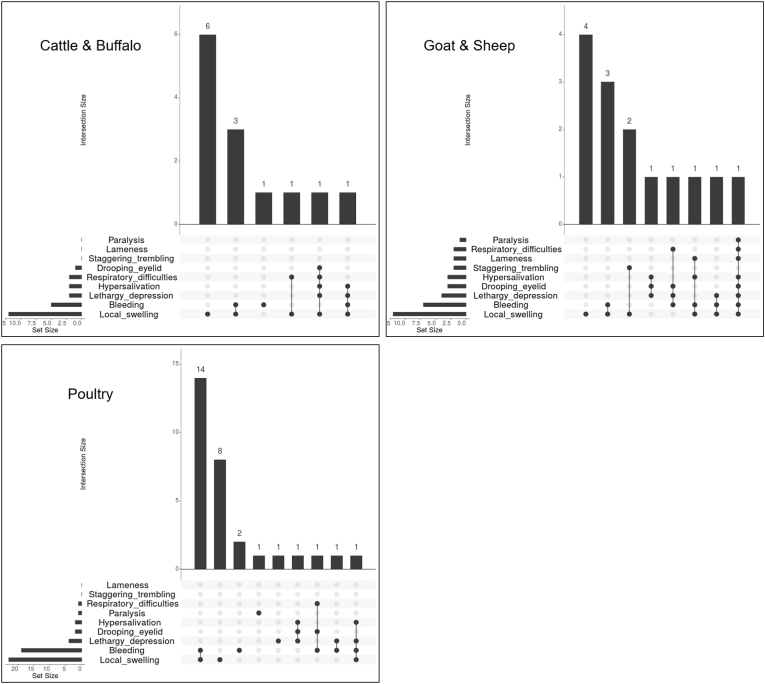
UpSet plots showing clinical signs of snakebite in cattle and buffalo, goat and sheep, and poultry in Nepal. The bar chart shows the number of snake-bitten animals that exhibited some particular combination of clinical signs. Each bar is a different combination. The graphical table underneath shows what those combinations are. The bar chart to the left of the graphical table accounts for the frequency of each clinical sign.

**Fig. 2:**
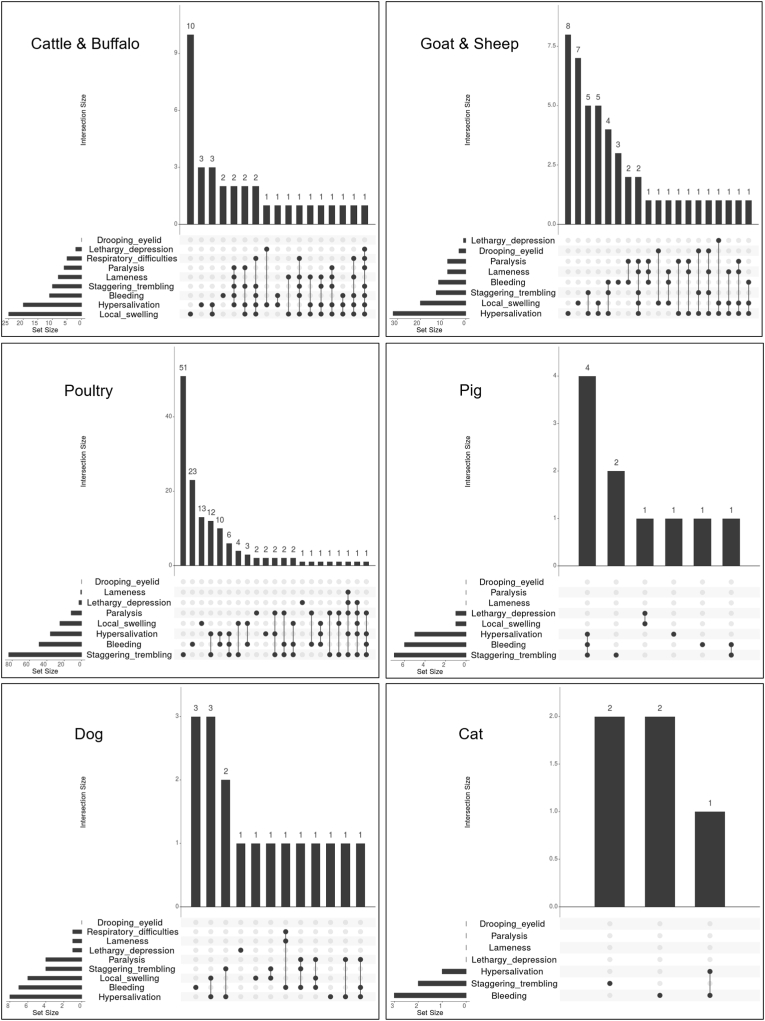
UpSet plots showing clinical signs of snakebite in cattle and buffalo, goat and sheep, poultry, pig, dog, and cat in Cameroon. The bar chart shows the number of snake-bitten animals that exhibited some particular combinations of clinical signs. The graphical table underneath shows what those combinations are. The bar chart to the left of the graphical table accounts for the frequency of each clinical sign.

A diversity of clinical signs were reported in animals affected by a snakebite. The distribution of these signs with their co-occurrence is shown in Fig. 1 for Nepal and Fig. 2 for Cameroon. In Nepal, the clinical signs most commonly observed in all types of animals were local swelling alone or combined with bleeding (Fig. 1). The animal victims also showed diverse combinations of other signs like lethargy-depression, drooping eyelids, hypersalivation, and respiratory difficulties. The case of snakebite in a dog showed bleeding, lethargy-depression, paralysis, and hypersalivation.

In Cameroon, besides local swelling and bleeding, other signs like hypersalivation and staggering-trembling were also commonly reported, with approximately half of the animals showing only one or two of these four signs (Fig. 2). A large diversity of clinical sign combinations was observed in the other half of the animals. In the two horse/donkey cases local swelling alone or combined with bleeding were reported.

Hypersalivation and respiratory difficulties could result either from pharyngeal paralysis due to a neurotoxic envenomation or from upper airway obstruction caused by excessive swelling following a bite in the head and neck region as reported in cattle, goats, and dogs (Leisewitz et al., 2004; Smith et al., 2015; Altuğ and İşler, 2019). We examined the site of the bite of the 11 animals in Nepal having at least one of these two signs. They were bitten on a limb (n = 6), the thorax-abdomen (n = 2), the head and neck area (n = 1), or on an unknown site (n = 2). In Cameroon, the 104 animals showing hypersalivation and/or respiratory difficulties were bitten on a limb (n = 47), head and neck area (n = 20), thorax-abdomen (n = 13), tail (n = 8), or on an unknown site (n = 16).

In both countries, owners reported clinical signs other than the options provided in the questionnaire, particularly in poultry. In Nepal, they observed a dark blue colour developed in the whole body (n = 5), bleeding and necrosis at the bite site (n = 2), a yellowish appearance (n = 1), or restlessness (n = 1). In Cameroon, other clinical signs in poultry included a black colour of the bitten spot (n = 22), a brick red colour of the skin (n = 4), a blood clot in the bitten area (n = 2), or the sudden death of the animal victim (n = 12). Vomiting was reported in sheep (n = 1) and dogs (n = 2), and urination in a cat.

In seven poultry cases in Nepal and eight in Cameroon (including seven chicks), no clinical sign was reported because the animals were eaten by the snake (i.e. predation).

### 3.3 Treatment

A total of six (8%) animal victims in Nepal and 31 (10%) in Cameroon received some kind of treatment after a snakebite. The only types of animals to be treated were ruminants and dogs. Cattle and buffaloes were the animals most frequently treated, as 40% of them in Nepal and 51% in Cameroon received treatment. The treatment modalities of animal victims are shown in Table 3.

**Table 3:** Types of treatment given to animals in Nepal and Cameroon after a snakebite.

Treated by/Types of treatment	Animal victims treated in Nepal			Animal victims treated in Cameroon			
	Cattle & Buffalo (n = 4) n(%)	Goat & Sheep (n = 2) n(%)	Total (n = 6) n(%)	Cattle & Buffalo (n = 18) n(%)	Goat & Sheep (n = 7) n(%)	Dog (n = 6) n(%)	Total (n = 31) n(%)
**By a traditional healer**	2 (50)	1 (50)	3 (50)	12 (67)	2 (29)	2 (33)	16 (52)
with herbs	2	1	3	12	2	2	16
**By the owner/family member**	0 (0)	0 (0)	0 (0)	6 (33)	3 (43)	4 (67)	13 (42)
with herbs	0	0	0	3	0	0	3
with antibiotic injection	0	0	0	1	0	0	1
with bloodletting	0	0	0	0	1	2	3
with bloodletting/herbs/part of snake/antibiotic	0	0	0	0	1	0	1
with another treatment	0	0	0	2	1	2	5
**By a veterinarian/animal health service provider**	2 (50)	1 (50)	3 (50)	0 (0)	2 (29)	0 (0)	2 (6)
with antibiotic injection	0	1	1	0	1	0	1
with antivenom	0	0	0	0	0	0	0
with another treatment	2	0	2	0	1	0	1

In Nepal, a traditional healer or an animal health service provider administered the treatment, while in Cameroon this was predominantly done by a traditional healer, the animal owner, or an owner's family member (94% of snakebite cases) with rare involvement of a veterinarian (6% of animal cases). Traditional healers, owners, and family members used a diversity of traditional medicines (e.g., herbs, bloodletting). On the contrary, the veterinarian administered modern medicine (i.e. antibiotics). The animals were predominantly treated with herbs (n = 3, 50% in Nepal and n = 19, 61% in Cameroon). No animal received snake antivenom therapy after a snakebite.

### 3.4 Outcome

The vast majority of the animals died as a consequence of the bite (85% in Nepal, 87% in Cameroon) and 1% of the animals in Nepal and 4% in Cameroon were slaughtered (Table 4). Death was less frequent in cattle and buffaloes (73% in Nepal, 63% in Cameroon) than in other types of animals. The surviving animals either fully recovered (5% in Nepal and Cameroon) or recovered with lameness (1% in Cameroon) or the loss of a body part (0.3% in Cameroon). The number of animals that recovered without being treated (n = 7) was too small to assess the association between clinical signs and outcome.

**Table 4:** The outcome of snakebite in animals in Nepal and Cameroon.

Outcome of snakebite	Types of animals in Nepal					Types of animals in Cameroon							
	Cattle & Buffalo (n = 15) n(%)	Goat & Sheep (n = 14) n(%)	Poultry (n = 43) n(%)	Dog (n = 1) n(%)	Total (n = 73) n(%)	Cattle & Buffalo (n = 35) n(%)	Horse & Donkey (n = 2) n(%)	Goat & Sheep (n = 50) n(%)	Pig (n = 11) n(%)	Poultry (n = 207) n(%)	Dog (n = 19) n(%)	Cat (n = 8) n(%)	Total (n = 332) n(%)
Spontaneous death	11 (73)	13 (93)	37 (86)	1 (100)	62 (85)	22 (63)	2 (100)	42 (84)	6 (55)	199 (96)	12 (63)	7 (88)	290 (87)
Slaugthered	0 (0)	0 (0)	1 (2)	0 (0)	1 (1)	2 (6)	0 (0)	3 (6)	5 (45)	2 (1)	2 (11)	0 (0)	14 (4)
Full recovery	3 (20)	1 (7)	0 (0)	0 (0)	4 (5)	10 (29)	0 (0)	5 (10)	0 (0)	0 (0)	2 (11)	0 (0)	17 (5)
Recovery with loss of body part	0 (0)	0 (0)	0 (0)	0 (0)	0 (0)	0 (0)	0 (0)	0 (0)	0 (0)	0 (0)	1 (5)	0 (0)	1 (0)
Recovery with lameness	0 (0)	0 (0)	0 (0)	0 (0)	0 (0)	1 (3)	0 (0)	0 (0)	0 (0)	0 (0)	1 (5)	0 (0)	2 (1)
Other	0 (0)	0 (0)	5 (12)	0 (0)	5 (7)	0 (0)	0 (0)	0 (0)	0 (0)	6 (3)	0 (0)	0 (0)	6 (2)
Unknown	1 (7)	0 (0)	0 (0)	0 (0)	1 (1)	0 (0)	0 (0)	0 (0)	0 (0)	0 (0)	1 (5)	1 (13)	2 (1)

In Nepal, two out of the six (33%) animals that were treated survived, and 15 out of 31 (48%) in Cameroon. We investigated the association between snakebite treatment and outcome in ruminants in Cameroon. Among the ruminants with a snakebite that were not slaughtered (n = 80), 24 had been treated (either with herbs (16), antibiotic (2), bloodletting (1), a combination of treatments (1) or an unknown treatment (4)). These animals were more likely to recover than those that were not treated (50% *vs* 7%, OR = 12.44 [95% CI 3.53–51.74]). This result was also significant when taking into account only the ruminants treated with herbs (OR = 12.3 [95% CI 3.04–57.07]). To explore the possibility that treated animals recovered because they had fewer clinical signs (e.g., only local swelling) we compared the number of clinical signs in treated and non-treated ruminants; 46% treated ruminants and only 27% non-treated ruminants showed three or more clinical signs. Thus, the treatment, and especially herbs, had a significant effect on the recovery of ruminants from snakebite even when those treated had on average more clinical signs. The number of animals treated in Nepal was too small to do this analysis.

## 4 Discussion

To our knowledge, this is the first nation-wide and community-based snakebite study that comprehensively describes clinical signs, treatments, and health outcomes of snakebite in animals in endemic LMICs in Asia (Nepal) and sub-Saharan Africa (Cameroon). The breadth of this study, including 405 cases of snakebite in 10 types of animals, is unprecedented. A wide diversity of clinical manifestations, similar to those often described in humans, was reported in all animal groups with an overall mortality rate of 85% in Nepal and 87% in Cameroon. Only 8% of animal victims in Nepal and 10% in Cameroon, mainly ruminants and dogs, were treated with traditional or modern medicine.

### 4.1 Scene of the snake bites and prevention of snakebite in livestock

We found that the snake bite in animals mainly occurred inside or around the house or farm, which coincides with snakebite cases in humans. For example, in South-East Asia, 59.2% of snake bite in humans occurs inside and around human habitations (WHO, 2016). In Cameroon, and to a smaller extent in Nepal, field and pasture were also common locations of snakebite to animals (in 40% of cattle and buffaloes cases and 34% of goat and sheep cases). Snakes were seen by livestock herders in these snakebite cases, which also puts them at risk of snakebite while accompanying their animals in the field. In fact, epidemiological studies of snakebite in humans in Cameroon (Chippaux et al., 2002; Einterz and Bates, 2003) and other sub-Saharan African countries (e.g., Kenya, Nigeria, and Tanzania) (Warrell and Arnett, 1976; Habib et al., 2001; Kipanyula and Kimaro, 2015; Ochola et al., 2018) showed that pastoralist activities are often associated with snake encounters and snakebite.

Prevention of snakebite in livestock can be improved by underpinning production practices with a better understanding of the behaviour of locally present snakes. Measures such as a regular check of barns for rodents and snakes, rodent population management, regular cleaning of the barns and surrounding areas, including removing of any waste food, keeping the grass short or any vegetation surrounding animal premises, and using light when an animal is walked out of the barn at night could be important in the prevention of snakebite in livestock (Joshi et al., 2018). Such prevention practices are particularly relevant during flooding events, the rainy season, and harvesting seasons (Joshi et al., 2018; Bolon et al., 2019; Ochoa et al., 2020). Importantly, such measures can reduce snakebite risk in both people and their animals (WHO, 2016).

Household poultry farming seems particularly problematic because hens, chicks, and eggs are potential prey for snakes (e.g., *Naja* sp). Most snakebite cases were reported in poultry in both study countries, with farmers often witnessing snakes swallowing chicks and hens. Lawal et al. described 21 cases of snakebite in poultry involving five farms in Nigeria. Hens incubating eggs were the most affected, and eggs and chicks were predated (Lawal et al., 1992). The responsible snake was suspected to be a cobra. In Maharashtra in India, attack by snakes was the second most frequent problem reported by a sample of 240 backyard poultry owners (Khandait et al., 2011). Poultry production should therefore consider the presence of snakes and include risk-mitigation strategies such as securing the coop with a narrow mesh, raising the floor of the coop, and frequently collecting eggs.

Raising snakebite awareness in farmers, particularly small-holders, including information on the behaviour of relevant snake species as well as information generated by studies such as this namely on seasonality and scene of the bite, will be key in prevention programs for snakebite in livestock.

### 4.2 Part of the body bitten specific to the type of animals

Cattle and buffaloes were mostly bitten on the limbs in both Nepal and Cameroon. This is in agreement with a study of 98 cattle bitten by snakes in India (Bhikane et al., 2020) and suggests that most often the bite happens when the animal accidently stepped on the snake. Since goats, sheep, and pigs are smaller animals, they were also bitten on the abdomen and thorax. There are few case reports on snakebite in small ruminants, but bites to these body parts were also reported in sheep in Brazil (Mendez and Riet-Correa, 1995) and goats in India and the US (Smith et al., 2015; Sivaraman et al., 2016). The head, limbs, or thorax were bitten in poultry as reported by Lawal et al. (1992). The predominance of bites to the head in dogs, which may result from their inquisitive behaviour, and to limbs in cats is in agreement with previous studies (Bolon et al., 2019).

### 4.3 Diversity of clinical signs

The clinical features of snakebite observed in animals were diverse in Nepal and Cameroon. The fact that snakebite data was collected across the country could explain this diversity. Nepal and Cameroon have a rich snake fauna with 14 medically important venomous snake species in Nepal and 17 in Cameroon (WHO, 2010). These species include both elapid and viperid snakes like cobras (*Naja* spp.) and kraits (*Bungarus* spp.) found throughout Nepal's Terai, and Russel's viper (*Daboia russelii*) occurring in southern Terai (Sharma et al., 2013). The forest cobras (*Naja melanoleuca*) and Gaboon vipers (*Bitis gabonica*) are particularly abundant in south-west Cameroon, and the northern region is dominated by the carpet viper (*Echis ocellatus*) (Gonwouo et al., 2005).

Snake venoms are highly diverse and their composition varies between species (Casewell et al., 2020). Viperid venom predominantly causes cytotoxicity (progressive swelling and tissue necrosis) and hemotoxicity (haemorrhage and coagulopathy), whereas elapid venom causes neurotoxicity (progressive paralysis) (Leisewitz et al., 2004; Gilliam and Brunker, 2011; Gutiérrez et al., 2017). Local swelling and bleeding were often observed in envenomed animals in our study, as it was also the case for cattle and goats envenomed by viperid snakes in India (Arul et al., 2020; Bhikane et al., 2020; Venkatesakumar et al., 2020). We also found clinical features suggesting neurotoxic bites (e.g., neurological signs like ptosis, paralysis, and hypersalivation or respiratory difficulties not associated with a bite on the head-neck region). Neurotoxic envenoming is poorly documented in livestock in LMICs, but similar neurological signs have been reported in two cows with suspected cobra bites, one in Pakistan and one in India (Farooq et al., 2014; Kachhawa et al., 2016). In addition, envenoming signs can vary between snake species of the same family (Viperidae or Elapidae). For instance, in Australia, where all medically important venomous snakes are elapids, a range of clinical syndromes has been reported for envenomed dogs, cats, and horses depending on the snake species (Padula et al., 2016). The body part bitten and the quantity of venom injected may also explain the diversity of signs reported.

### 4.4 The challenge of treating snakebite in animals

Owners sought treatment for a limited number of snakebite cases, particularly for ruminants (e.g., 40% of cattle and buffalo cases in Nepal and 51% in Cameroon) and dogs, and mainly relied on traditional medicine (e.g., herbs). Traditional medicine is often the only option to treat snakebite because livestock owners have limited access to veterinary healthcare services. In addition, antivenoms are in short supply in Nepal and Cameroon, particularly in rural areas, and too costly for often-impoverished farmers (Einterz and Bates, 2003; Difo et al., 2005; Shrestha et al., 2017; Tchoffo et al., 2019).

Traditional medicinal plants have been used to manage snakebite envenoming in livestock across South-East Asia (Phondani et al., 2010; Malla and Chhetri, 2012; Shrivastava et al., 2017; Suroowan et al., 2017; Ullah et al., 2017) and sub-Saharan Africa (Ismaila and Adamu, 2012; Tamiru et al., 2013; Setlalekgomo, 2015; Dinbiso et al., 2020; Dzoyem et al., 2020). We found an association between treatment with herbs and snakebite recovery in ruminants, yet, cases were collected retrospectively so subject to numerous biases and potential confounding factors, like the severity of the envenomation. Although ethno-veterinary medicine (i.e. the scientific term for traditional animal health care) has been used for centuries (Menegesha, 2020) and many medicinal plants have shown antivenom effects in vitro and in mice (Yirgu and Chippaux, 2019), the use of these traditional practices lacks scientific validation with prospective and randomized studies (WHO, 2013).

Antivenoms are currently the only effective and scientifically validated antidotes for snake venom. They have been used successfully to treat snakebite in cattle in Costa Rica (Rodriguez et al., 2016), and cattle, goats, and sheep in India (Kachhawa et al., 2016; Sivaraman et al., 2016; Senthilkumar et al., 2018; Sakhare et al., 2019; Ali and Arul, 2020; Arul et al., 2020; Bhikane et al., 2020; Venkatesakumar et al., 2020). However, the time between the bite and antivenom administration is critical and affects treatment efficacy in animals (Rodriguez et al., 2016; Bhikane et al., 2020). In Costa Rica, a significant number of *Bothrops asper* bites in cattle occurred at night (Rodriguez et al., 2016; Herrera et al., 2017), and cattle often die within hours after bites. This has prompted the development of a toxoid vaccine that extends the window for antivenom administration and increases the likelihood of survival (Herrera et al., 2017).

Therefore, when veterinary services and antivenoms are available, bitten animals should be rapidly brought to veterinary attention. However, lack of access to safe and effective antivenoms is a major issue in LMICs, and one of the priorities of the WHO global strategy for snakebite is to address this antivenom supply crisis (WHO, 2019). The issue of access to antivenoms in the veterinary sector in LMICs should be addressed in parallel or once access for humans is secured. The centralization of antivenoms for animal care in one location per district instead of having vials in many centers may facilitate stock management. The possibility of an exceptional dispensation for using expired antivenoms in animals if no other supplies are available and instead of discarding vials after expiry in hospitals could also be explored. In vitro studies showed that expired antivenoms maintain their activity for up to 15–20 years after their expiry date (O'Leary et al., 2009, Sánchez et al., 2019). They have been used to treat envenomed dogs and cats in Australia, the UK, and Switzerland with no increased side effects (Swindells et al., 2006; Sim, 2014; Fuchs et al., 2017).

Promising novel therapies (i.e. new molecules, toxin antagonists or inhibitors) are being developed to counteract or delay the pathological effects of venoms (Albulescu et al., 2020; Gutiérrez et al., 2020). They are especially interesting as they may be administered orally to victims in the absence of trained healthcare staff. They could be made available for veterinary medicine before human medicine because approval and registration of pharmaceutical products are faster in animal than human health (Delaveau, 2011).

### 4.5 High mortality due to snakebite

We found high mortality in envenomed livestock in Nepal and Cameroon. Our global scoping review showed mortality due to snakebite above 47% in half of the publications on snakebite in livestock (Bolon et al., 2019). Lawal et al. reported mortality of 87.5% in envenomed poultry in Nigeria (Lawal et al., 1992). These animal losses may have an important impact on the livelihood of farmers because herd size is often small and these rural communities are strongly dependent on their animals for food provision and income generation (Chapman et al., 1998; Otte and Chilonda, 2002; Maltsoglou and Taniguchi, 2004). We are currently quantifying this economic impact in Nepal and our preliminary results show that most households with animal snakebite cases reported livelihood losses. Depending on the animal type and production function, reported losses represent a significant proportion of the income of rural Nepali households and even surpass the average monthly earnings for rural households in the case of large ruminants.

Finally, the suspected snakebite cases collected in the *Snake-byte* project were not included in this study. However, their number (n = 322) illustrates that snakebite in animals is certainly underestimated and that more research and veterinary diagnostic capacities are required to assess the true burden of snakebite in animals.

### 4.6 Study limitations

This study has several limitations. First, data on snakebite in animals was collected retrospectively through a cross-sectional method, so is subject to recall bias and we did not validate the responses given by the owners. Yet, this potential bias was minimized by the short period studied (previous 12 months). Also, snakebite in livestock animals is a significant event when the herd size is small and there is a risk of animal death and income loss. However, we cannot exclude that owners may have preferentially recalled snakebite cases in animals when the impact on animal health was severe. This could explain the high mortality attributed to snakebite in animals. Second, we provided predefined responses for clinical signs, which may have predisposed the response of the interviewees. However, for each question, we also offered the possibility for the interviewee to add further comments to complement the predefined options. This may have minimized this potential bias. Third, the clinical signs were reported by the owners and could not be verified by veterinary examinations. However, the clinical signs proposed in the questionnaire (e.g., swelling, bleeding, lameness, etc.) are signs easy to visualize in animals in general (Joshi et al., 2018). Fourth, we cannot exclude that some clinical signs may have been missed by the owner either because they have gone unnoticed (e.g., ptosis, respiratory difficulties) or occurred at night in a dying animal (e.g., paralysis caused by a neurotoxic bite). In addition, we did not ask owners to report the extent of the swelling, which could be a clinical criterion for snakebite severity as reported in horses (Tirosh-Levy et al., 2017).

## 5 Conclusion

Snakebite in domestic animals is a significant yet neglected problem in LMICs. This countrywide community-based study comprehensively describes clinical signs, treatments, and health outcomes of snakebite in ten types of animals in Nepal and Cameroon. We showed that snakebite causes a diversity of clinical manifestations in all types of livestock animals and high mortality. Owners rarely seek treatment for animals bitten by snake and predominantly use traditional medicine, probably due to the lack of antivenoms or their high costs. There is a need for snakebite prevention strategies and affordable therapies in the veterinary field because snake antivenom production is currently prioritized for human use. Finally, this study highlights snakebite as a health problem occurring at the human-snake-domestic animal interface in endemic countries. Snakebite preventive measures should take into account the entire rural agro-ecosystem, including humans, domestic animals, and snakes using a systemic One Health approach. This aligns with the WHO global strategy for prevention and control of snakebite who emphasizes the need for a One Health approach to snakebite (WHO 2019).

## Credit author statement

Isabelle Bolon: Conceptualization, Methodology, Validation, Formal analysis, Investigation, Writing - Original Draft, Writing - Review & Editing; Sara Babo Martins: Conceptualization, Methodology, Validation, Investigation, Project administration, Writing - Review & Editing; Carlos Ochoa: Conceptualization, Methodology, Validation, Investigation, Data Curation, Writing - Review & Editing; Gabriel Alcoba: Conceptualization, Methodology, Validation, Investigation, Data Curation, Writing - Review & Editing; Maria Herrera: Methodology, Writing - Review & Editing; Henri Magloire Bofia Boyogueno: Validation, Writing - Review & Editing; Barun Sharma: Validation, Writing - Review & Editing; Manish Subedi: Methodology, Validation, Investigation, Writing - Review & Editing; Bhupendra Shah: Methodology, Validation, Investigation, Writing - Review & Editing; Franck Wanda: Conceptualization, Investigation, Methodology, Project administration, Writing - Review & Editing; Sanjib K. Sharma: Conceptualization, Methodology, Validation, Investigation, Writing - Review & Editing; Armand S. Nkwescheu: Conceptualization, Methodology, Validation, Investigation, Writing - Review & Editing; Nicolas Ray: Conceptualization, Methodology, Validation, Investigation, Resources, Writing - Review & Editing, Supervision, Project administration, Funding acquisition ; François Chappuis: Conceptualization, Methodology, Validation, Investigation, Resources, Writing - Review & Editing, Supervision, Project administration, Funding acquisition; Rafael Ruiz de Castañeda: Conceptualization, Methodology, Validation, Investigation, Writing - Review & Editing. Recommendations

• Academic researchers and public health authorities should acknowledge and further assess the communities' capabilities at identifying snakebite in domestic animals. Participatory research involving animal owners could generate new and contextualized knowledge for improved and more easily applied snakebite preventive measures.
• Educational programs on primary prevention of snakebite should take a systemic One Health approach and consider both human and animal exposure to snakes and snakebite risk. This will minimize the risk of snakebite in all species.
• Epidemiological research on snakebite in humans should collect data on the circumstances of the bite more granularly (e.g., refine “agricultural activities” or “farming”). This could help better understand the type of human-domestic animal interface and interactions that lead to human-snake encounters, and identify new primary preventive measures of snakebite for both animals and humans.
• Enhancing production systems with prevention measures for snakebite and better access to snake antivenoms, ancillary treatments, and novel therapies (specific antagonists, monoclonal antibodies) to treat snakebite in animals in LMICs could save animal lives, improve health outcomes and prevent livelihood losses. In line with the WHO global strategy for snakebite, national and regional human and veterinary health authorities should work together for better integration of animal health in the sub-national or national health plans for snakebite prevention and control.

## References

[bib1] Albulescu L.-O., Xie C., Ainsworth S., Alsolaiss J., Crittenden E., Dawson C.A., Softley R., Bartlett K.E., Harrison R.A., Kool J. (2020). A therapeutic combination of two small molecule toxin inhibitors provides broad preclinical efficacy against viper snakebite. Nat. Commun..

[bib2] Alcoba G., Ochoa C., Babo Martins S., Ruiz de Castañeda R., Bolon I., Wanda F., Comte E., Subedi M., Shah B., Ghimire A., Gignoux E., Luquero F., Nkwescheu A.S., Sharma S.K., Chappuis F., Ray N. (2021). Novel transdisciplinary methodology for cross-sectional analysis of snakebite epidemiology at national scale. PLoS Neglected Trop. Dis..

[bib3] Ali M.S., Arul A.R.V. (2020). Successful medical management of snake envenomation in a Jersey crossbred cow. J. Entomol. Zool. Stud..

[bib4] Altuğ N., İşler C.T. (2019). Snake envenomation in two cattle: clinical/laboratory aspects and treatment using equine-derived antivenin of Viperidae. Turk. J. Vet. Anim. Sci..

[bib5] Anlén K.G. (2008). Effects of bites by the European adder (Vipera berus) in seven Swedish horses. Vet. Rec..

[bib6] Arul V., Inbaraj C., Brindha V. (2020). Management of snake bite envenomation in an indigenous cow. J. Entomol. Zool. Stud..

[bib7] Babo Martins S., Bolon I., Chappuis F., Ray N., Alcoba G., Ochoa C., Kumar Sharma S., Nkwescheu A.S., Wanda F., Durso A.M. (2019). Snakebite and its impact in rural communities: the need for a one health approach. PLoS Neglected Trop. Dis..

[bib8] Bamford N., Sprinkle S., Cudmore L., Cullimore A., Van Eps A., Verdegaal E., Tennent- Brown B. (2018). Elapid snake envenomation in horses: 52 cases (2006–2016). Equine Vet. J..

[bib9] Bhikane A., Jadhav R., Masare P., Chavhan S. (2020). Clinical, hematobiochemical, and pathological findings and therapeutic management of viperine snake envenomation in zebu cattle. Trop. Anim. Health Prod..

[bib10] Bolon I., Finat M., Herrera M., Nickerson A., Grace D., Schütte S., Martins S.B., de Castañeda R.R. (2019). Snakebite in domestic animals: first global scoping review. Prev. Vet. Med..

[bib11] Casewell N.R., Jackson T.N., Laustsen A.H., Sunagar K. (2020). Causes and consequences of snake venom variation. Trends Pharmacol. Sci..

[bib12] Chapman D.R., Joshi D.D., Joshi Y.R. (1998). A Survey of the Snake-Bite Problem in Humans and Livestock in the Terai Region of Nepal. https://libraryopac.searo.who.int/cgi-bin/koha/opac-detail.pl?biblionumber=3806.

[bib13] Chippaux J.-P., Rage-Andrieux V., Le Mener-Delore V., Charrondiere M., Sagot P., Lang J. (2002). Epidemiologie des envenimations ophidiennes dans le nord du Cameroun. Bull. Soc. Pathol. Exot..

[bib14] Cullimore A.M., Lester G.D., Swindells K.L. (2013). Tiger snake (Notechis scutatus) envenomation in a horse. Aust. Vet. J..

[bib15] Dean A., Sullivan K.M., Soe M.M. (2013). OpenEpi: Open Source Epidemiologic Statistics for Public Health. http://www.OpenEpi.com.

[bib16] Delaveau J. (2011). Differences in the registration of veterinary and human pharmaceutical products. Bull. Acad. Vet. Fr..

[bib17] Difo J.L.D., Dzikouk G., LeBreton M., Ngoa L.E., Chirio L., Moyou R.S. (2005). Distribution des sérums antivenimeux au Cameroun. Bull. Soc. Pathol. Exot..

[bib18] Dinbiso T.D., Tolosa T.T., Begna F.D. (2020). Ethnoveterinary Practices of Medicinal Plants and Non-plant Remedies Used in Animal Health Management in Dawuro Zone, Southern Ethiopia.

[bib19] do Rego Leal M.L., Aires A.R., Fillapi A., Trost M.E. (2013). Clinical and pathological observations associated with snake envenomation in two sheep. Acta Sci. Vet..

[bib20] Dzoyem J.P., Tchuenteu R.T., Mbarawa K., Keza A., Roland A., Njouendou A.J., Assob J.C.N. (2020). Ethnoveterinary medicine and medicinal plants used in the treatment of livestock diseases in Cameroon. Ethnoveterinary Medicine.

[bib21] Einterz E.M., Bates M.E. (2003). Snakebite in northern Cameroon: 134 victims of bites by the saw-scaled orcarpet viper, Echis ocellatus. Trans. R. Soc. Trop. Med. Hyg..

[bib22] FAO (2009). Livestock keepers: guardians of biodiversity. Food and Agriculture Organization of the United Nations.

[bib23] Farooq U., Irshad H., Ullah R.W., Ullah A., Afzal M., Latif A., Bin A. (2014). Snake bite in Jersey cattle; a case report. Res. J. Vet. Pract..

[bib24] Fielding C.L., Pusterla N., Magdesian K.G., Higgins J.C., Meier C.A. (2011). Rattlesnake envenomation in horses: 58 cases (1992–2009). J. Am. Vet. Med. Assoc..

[bib25] Fuchs J., Casado Diaz J.I., Jud Schefer R., Rauber-Lüthy C. (2017). Expired antivenom: good efficacy in a severely envenomed cat bitten by Sistrurus miliarius miliarius (Carolina Pigmy Rattlesnake). Clin. Toxicol..

[bib26] Gilliam L.L., Brunker J. (2011). North American snake envenomation in the dog and cat. Vet. Clin. N. Am. Small Anim. Pract..

[bib27] Gómez S.E., Castillo J.C.Q., Muñoz L.J.V. (2014). Accidente ofídico en animales de pastoreo: acercamiento epidemiológico, clínico y de manejo. Rev. Med. Vet..

[bib28] Gonwouo N.L., LeBreton M., Chirio L., Ngassam P., Ngoa L.E., Dzikouk G. (2005). Biogeographical distribution of snakes in Cameroon: the case of venomous snakes. Bull. Soc. Pathol. Exot..

[bib29] Gutiérrez J.M., Calvete J.J., Habib A.G., Harrison R.A., Williams D.J., Warrell D.A. (2017). Snakebite envenoming. Nat. Rev. Dis. Prim..

[bib30] Gutiérrez J.M., Lewin M.R., Williams D., Lomonte B. (2020). Varespladib (LY315920) and methyl varespladib (LY333013) abrogate or delay lethality induced by presynaptically acting neurotoxic snake venoms. Toxins.

[bib31] Habib A., Gebi U., Onyemelukwe G. (2001). Snake bite in Nigeria. Afr. J. Med. Med. Sci..

[bib32] Herrera M., González K., Rodríguez C., Gómez A., Segura Á., Vargas M., Villalta M., Estrada R., León G. (2017). Active immunization of cattle with a bothropic toxoid does not abrogate envenomation by Bothrops asper venom, but increases the likelihood of survival. Biologicals.

[bib33] Ismaila M., Adamu S. (2012). The impact of traditional methods of managing snake bite in humans and livestock among the Hausa-Fulani communities of Sokoto State (North-western Nigeria). J. Med. Plants Res..

[bib34] Joshi V., Alam S., Dimri U. (2018). Snake bites in farm animals: a field guide. Indian Dairym..

[bib35] Kachhawa J.P., Sharla A., Tanwar T.K., Singh A.P. (2016). Therapeutic management of snake bite in buffalo- A Case Report. Indian J. Vet. Med..

[bib36] Khandait V., Gawande S., Lohakare A., Dhenge S. (2011). Adoption level and constraints in backyard poultry rearing practices at Bhandara District of Maharashtra (India). Res. J. Agric. For. Sci..

[bib37] Kipanyula M., Kimaro W. (2015). Snakes and snakebite envenoming in Northern Tanzania: a neglected tropical health problem. J. Venom. Anim. Toxins Incl. Trop. Dis..

[bib38] Koscinczuk P., Acosta de Perez O., Teibler P., Marunak S., Rosciani A.S. (2000). American rattlesnake (Crotalus durissus terrificus) bite accidents in dogs in Argentina. Arq. Bras. Med. Vet. Zootec..

[bib39] Lawal S., Abdu P.A., Jonathan G.B., Hambolu O.J. (1992). Snakebites in poultry. Vet. Hum. Toxicol..

[bib40] Leisewitz A.L., Blaylock R.S., Kettner F., Goodhead A., Goddard A., Schoeman J.P. (2004). The diagnosis and management of snakebite in dogs-a southern African perspective: review article. J. S. Afr. Vet. Assoc..

[bib41] Lervik J.B., Lilliehook I., Frendin Jan H.M. (2010). Clinical and biochemical changes in 53 Swedish dogs bitten by the European adder - Vipera berus. Acta Vet. Scand..

[bib42] Lex A., Gehlenborg N., Strobelt H., Vuillemot R., Pfister H. (2014). UpSet: visualization of intersecting sets. IEEE Trans. Visual. Comput. Graph..

[bib43] Malla B., Chhetri R. (2012). Ethnoveterinary practices of some plant species by ethnic people of Parbat district, Nepal. Kathmandu Univ. J. Sci. Eng. Technol..

[bib44] Maltsoglou I., Taniguchi K., E.W.P.N. (2004). Poverty, livestock and household typologies in Nepal. http://www.fao.org/3/ae125e/ae125e00.htm.

[bib45] Mendez M.C., Riet-Correa F. (1995). Snakebite in sheep. Vet. Hum. Toxicol..

[bib46] Menegesha A. (2020). A review on ethno-veterinary medicine practices and indigenous knowledge. J. Vet. Med. Animal Sci..

[bib47] National Institute of Statistics and Census (2019). National Agricultural Survey: General Results of the Bovine and Porcine Farming Activity (Year 3). https://www.inec.cr/sites/default/files/documetos-biblioteca-virtual/reagropecenapecuario2019-01.pdf.

[bib48] Ochoa C., Bolon I., Durso A.M., Ruiz de Castañeda R., Alcoba G., Babo Martins S., Chappuis F., Ray N. (2020). Assessing the increase of snakebite incidence in relationship to flooding events. J. Environ. Public Health.

[bib49] Ochola F.O., Okumu M.O., Muchemi G.M., Mbaria J.M., Gikunju J.K. (2018). Epidemiology of snake bites in selected areas of Kenya. Pan Afr. Med. J..

[bib50] O'Leary M.A., Kornhauser R.S., Hodgson W.C., Isbister G.K. (2009). An examination of the activity of expired and mistreated commercial Australian antivenoms. Trans. R. Soc. Trop. Med. Hyg..

[bib51] Onoviran O., Olufemi B.E., Onunkwo O. (1976). Snakebite in a hen. Vet. Rec..

[bib52] Otte M., Chilonda P. (2002). Cattle and Small Ruminant Production Systems in Sub-saharan Africa. A Systematic Review. http://www.fao.org/3/a-y4176e.pdf.

[bib53] Padula A., Ong H., Kelers K. (2016). Snake envenomation in domestic animal species in Australia. Clinical Toxinology: Clinical Toxinology.

[bib54] Pham P., Vinck P., Kreutzer T., Milner J., Dorey A., Musaraj P. (2019). KoBoToolbox| Data Collection Tools for Challenging Environments. https://www.kobotoolbox.org.

[bib55] Phondani P., Maikhuri R., Kala C. (2010). Ethnoveterinary uses of medicinal plants among traditional herbal healers in Alaknanda catchment of Uttarakhand, India. Afr. J. Tradit., Complementary Altern. Med..

[bib56] Prasanth C., Khan S., Ajithkumar S. (2017). Therapeutic management of cobra envenomation in a goat. Intas Polivet.

[bib57] R-Core-Team. R (2020). A Language and Environment for Statistical Computing. http://www.r-project.org/index.html.

[bib58] Rodriguez C., Estrada R., Herrera M., Gomez A., Segura A., Vargas M., Villalta M., Leon G. (2016). Bothrops asper envenoming in cattle: clinical features and management using equine-derived whole IgG antivenom. Vet. J..

[bib59] Sakhare M., Siddiqui M., Shafi T., Borikar S., Pawar M., Shelke V. (2019). Clinical management of snake envenomation in a sheep-a case report. Indian J. Vet. Med..

[bib60] Sánchez E.E., Migl C., Suntravat M., Rodriguez-Acosta A., Galan J.A., Salazar E. (2019). The neutralization efficacy of expired polyvalent antivenoms: an alternative option. Toxicon.

[bib61] Segev G., Shipov A., Klement E., Harrus S., Kass P., Aroch I. (2004). Vipera palaestinae envenomation in 327 dogs: a retrospective cohort study and analysis of risk factors for mortality. Toxicon.

[bib62] Sen A., Chander M. (2003). Privatization of veterinary services in developing countries: a review. Trop. Anim. Health Prod..

[bib63] Senthilkumar A., Senthilrajaprabu R., Sribalaji N. (2018). Therapeutic management of snake envenomation in a crossbred dairy cattle-a case report. Res J. Chem. Environ. Sci..

[bib64] Setlalekgomo M.R. (2015). Snakebite management in cattle by farmers in lentsweletau extension area of Kweneng District in Botswana. Int. J. Innov. Res. Sci. Eng. Technol..

[bib65] Sharma S., Pandey D., Shah K., Tillack F., Chappuis F., Thapa C., Alirol E., Kuch U. (2013). Venomous Snakes of Nepal. A Photographic Guide.

[bib66] Shrestha B.R., Pandey D.P., Acharya K.P., Thapa-Magar C., Mohamed F., Isbister G.K. (2017). Effective, polyvalent, affordable antivenom needed to treat snakebite in Nepal. Bull. World Health Organ..

[bib67] Shrivastava S., Jain A.K., Tomar R.S. (2017). Ethnoveterinary practices: a review on phytotherapeutical approaches in treatment of animals. World J. Pharma. Med. Res..

[bib68] Sim A.B. (2014). Availability of antivenom for treating adder bites in dogs. Vet. Rec..

[bib69] Sivaraman S., Vijayakumar G., Venkatesakumar E., Ponnuswamy K. (2016). Russell's viper snake envenomation in a goat-A case report. Indian Vet. J..

[bib70] Smith J., Kovalik D., Varga A. (2015). Rattlesnake Envenomation in Three Dairy Goats. Case Reports in Veterinary Medicine 2015.

[bib71] Stewart G. (1974). Snakebite in pigs: a case report. Rhod. Vet. J..

[bib72] Swindells K.L., Russell N.J., Angles J.M., Foster S.F. (2006). Four cases of snake envenomation responsive to death adder antivenom. Aust. Vet. J..

[bib73] Suroowan S., Javeed F., Ahmad M., Zafar M., Noor M.J., Kayani S., Javed A., Mahomoodally M.F. (2017). Ethnoveterinary health management practices using medicinal plants in South Asia–a review. Vet. Res. Commun..

[bib74] Sutton N.M., Bates N., Campbell A. (2011). Canine adder bites in the UK: a retrospective study of cases reported to the Veterinary Poisons Information Service. Vet. Rec..

[bib75] Tamiru F., Terfa W., Kebede E., Dabessa G., Roy R.K., Sorsa M. (2013). Ethnoknowledge of plants used in veterinary practices in Dabo Hana district, West Ethiopia. J. Med. Plants Res..

[bib76] Tchoffo D., Kamgno J., Kekeunou S., Yadufashije C., Djeunga H.C.N., Nkwescheu A.S. (2019). High snakebite underreporting rate in the Centre Region of Cameroon: an observational study. BMC Publ. Health.

[bib77] Tirosh-Levy S., Solomovich R., Comte J., Sutton G.A., Steinman A. (2017). Daboia (vipera) Palaestinae envenomation in horses: clinical and hematological signs, risk factors for mortality and construction of a novel severity scoring system. Toxicon.

[bib78] Tokarnia C.H., Peixoto P.V. (2006). The importance of snake bites as cause of cattle death in Brazil. Pesqui. Vet. Bras..

[bib79] Turkovic V., Teichmann S., Dorfelt R. (2015). European Adder bites in dogs in southern Germany. A retrospective study over a 6.5-year period. Tierarztl. Prax. Ausg. K Kleintiere Heimtiere.

[bib80] Ullah Z., Shah G.M., Muhammad S., Muhammad Z., Ullah R., Majeed A. (2017). Ethnoveterinary plants used for animal cure in district Charsadda, Khyber Pakhtunkhwa (Pakistan). Spec J. Biol. Sci..

[bib81] Van den Bossche P., Thys E., Elyn R., Marcotty T., Geerts S. (2004). The provision of animal health care to smallholders in Africa: an analytical approach. OIE Rev. Sci. Tech..

[bib82] Venkatesakumar E., Sivaraman S., Vijayakumar G., Senthilkumar G., Mohanambal K., Ravi R. (2020). Therapeutic management of saw scaled viper (Echis carinatus) snake envenomation in a goat. J Entomo. Zool. Stud..

[bib83] Warrell D.A., Arnett C. (1976). The importance of bites by the saw-scaled or carpet viper (Echis carinatus): epidemiological studies in Nigeria and a review of the world literature. Acta Trop..

[bib84] WHO (2010). Venomous Snakes Distribution and Species Risk Categories. https://apps.who.int/bloodproducts/snakeantivenoms/database/.

[bib85] WHO (2013). WHO Traditional Medicine Strategy: 2014-2023. WHO Traditional Medicine Strategy: 2014-2023. https://www.who.int/medicines/publications/traditional/trm_strategy14_23/en/.

[bib86] WHO (2016). Regional Office for South-East Asia, Guidelines for the Management of Snakebite. https://apps.who.int/iris/handle/10665/249547.

[bib87] WHO (2019). Snakebite Envenoming: a Strategy for Prevention and Control. https://scholar.google.ch/scholar?hl=en&as_sdt=0%2C5&q=Snakebite+Envenoming%3A+a+Strategy+for+Prevention+and+Control&btnG=.

[bib88] Witsil A.J., Wells R.J., Woods C., Rao S. (2015). 272 cases of rattlesnake envenomation in dogs: demographics and treatment including safety of F(ab’)2 antivenom use in 236 patients. Toxicon.

[bib89] Yirgu A., Chippaux J.-P. (2019). Ethnomedicinal plants used for snakebite treatments in Ethiopia: a comprehensive overview. J. Venom. Anim. Toxins Incl. Trop. Dis..

