# Corrigendum to “What is the impact of snakebite envenoming on domestic animals? A nation-wide community-based study in Nepal and Cameroon” [Toxicon: X 9 (2021) 100068]

**DOI:** 10.1016/j.toxcx.2022.100133

**Published:** 2022-07-11

**Authors:** Isabelle Bolon, Sara Babo Martins, Carlos Ochoa, Gabriel Alcoba, María Herrera, Henri Magloire Bofia Boyogueno, Barun Kumar Sharma, Manish Subedi, Bhupendra Shah, Franck Wanda, Sanjib Kumar Sharma, Armand Seraphin Nkwescheu, Nicolas Ray, François Chappuis, Rafael Ruiz de Castañeda

**Affiliations:** a Institute of Global Health, Department of Community Health and Medicine, Faculty of Medicine, University of Geneva, Chemin des mines 9, 1202, Geneva, Switzerland; b Institute for Environmental Sciences, University of Geneva, 66 boulevard Carl-Vogt, 1205, Geneva, Switzerland; c Division of Tropical and Humanitarian Medicine, Geneva University Hospitals (HUG), Department of Community Health and Medicine, Faculty of Medicine, 24 rue Micheli-du-Crest, Geneva 14, 1211, Switzerland; d Médecins Sans Frontières (MSF), Rue de Lausanne 78, 1202, Geneva, Switzerland; e Instituto Clodomiro Picado, Facultad de Microbiología, Universidad de Costa Rica, San José, 11501-2060, Costa Rica; f Ministère de l’Elevage, des Pêches et des Industries Animales (MINEPIA), Direction des Services Vétérinaires, Yaoundé, Cameroon; g Ministry of Agriculture and Livestock Development, Singhadurbar, Kathmandu, Nepal; h B.P. Koirala Institute of Health Sciences (BPKIHS), Buddha Road, Dharan, 56700, Nepal; i Centre International de Recherche, d’Enseignement et de Soins en Milieu Tropical (CIRES), BP 11, Akonolinga, Cameroon; j Cameroon Society of Epidemiology (CaSE), P.O.Box 1411, Yaoundé, Cameroon; k Faculty of Medicine and Biomedical Science, University of Yaoundé 1, Yaoundé, Cameroon

The authors declare that they have no known competing financial interests or personal relationships that could have appeared to influence the work reported in this paper.

