# Supplementary material for: What is the impact of snakebite envenoming on domestic animals? A nation-wide community-based study in Nepal and Cameroon
Source: Toxicon X. 2021 Jun 5;9-10:100068. doi: 10.1016/j.toxcx.2021.100068 (PMC8214143; doi:10.1016/j.toxcx.2021.100068)
Supplement: Multimedia component 1 [file mmc1.docx]

Bolon I, Babo Martins S, Ochoa C, et al. What is the impact of snakebite envenoming on domestic animals? A nation-wide community-based study in Nepal and Cameroon

*Toxicon:X Special Issue: "A Transdisciplinary Approach to Snakebite Envenoming"*

**Supplemental information**

***Snake-Byte* Project
Survey Questionnaire
Animal Part**

**Bloc [3] Questions in case of Animal losses:
*- to complete if basic consent form is filled and signed by the head of household***

1. What animal was bitten? And how many (number per species)

- Cattle/buffalo ___
- Horse/donkey ___
- Goat ___
- Sheep ___
- Pig ___
- Poultry ___
- Camel ___
- Dog ___
- Cat ___
- Other ___

1. How did you conclude that the animal was bitten by a snake?
   1. Because you, or another person, witnessed the bite
   2. Because the animal has been seen interacting with a snake (playing, fighting), a live/dead snake was found close to the animal
   3. Because you found fang marks on the animal
   4. Because you observed specific envenoming signs
   5. Because you found the animal dead
   6. Other _____
2. **If yes to a or b,** did you identify the snake? *See list of local names in Nepal and Cameroon*
   - Name ____________
3. **If yes to c**, what was the site of the bite?

- head-face-neck
- limbs
- belly-back-shoulder
- udder

1. **If yes to d**, which were the signs observed that lead you to say it was a snakebite?

- local swelling (face, limb, other site)
- bleeding from eyes, ears, nose, gingiva, skin, presence of bloody stool
- staggering and trembling
- lethargy, depression
- lameness
- recumbency, the animal is unable to stand, is paralysed
- drooping of the eyelid
- hypersalivation
- respiratory difficulties
- other signs
- the animal was bitten by a snake but had no health issues

1. **If yes to e**, which were the signs observed that lead you to say it was a snakebite?

- local swelling (face, limb, other site)
- Bleeding (from eyes, ears, nose, gingiva, skin, blood in stool)
- Hypersalivation
- Other signs

1. In which month was your animal bitten?

- Don’t know
- ---------------(month)

1. Where did the bite to the animal take place?

- don’t know
- in the house/farm
- around the house/farm
- in a pen/stall/coop/stable
- in the field/pasture
- in a plantation
- close to a river
- in a forest
- other

1. Did you treat/seek treatment for the animal?

- NO
- YES

1. Who treated the animal?

- You or a family member
- a traditional healer
- a veterinarian

1. What type of treatment?

- bloodletting (saignée)
- herbs/plants
- use of part of the snake, snake bile
- drinking milk or other liquid
- antivenom
- antibiotic injection
- steroids
- other

1. Have you spent money on treatment expenses for your animal?

- No
- Yes, on modern veterinary medicine -> how much? ____ Rs/ CFA per animal head
- Yes, on traditional medicine -> how much? ____ Rs/ CFA per animal head

1. What was the outcome of the animal bitten? (Multiple answers possible)

- Found dead
- Found alive but finally died
- Slaughtered due to bite and buried
- Slaughtered due to bite and meat consumed
- Slaughtered due to bite and meat sold for normal price
- Slaughtered due to bite and meat sold for salvage price
- Sold for the normal price
- Sold for a cheaper (salvage) price
- Abortion
- Full recovery with no sequelae
- Recovery with loss of body part (ear, limb, ..)
- Recovery with infection-abscess
- Recovery with lameness
- Other

1. If the animal recovered after the bite, has there been changes in its productivity?

- No
- I don’t know
- Decreased milk productivity_ average milk lost per day ( L/day) and days of reduced productivity
- Decreased fertility_ Offspring lost/per reproduction season
- Decreased carcass weight at slaughter _ Kg
- Decreased market value of the live animal sold
- Decreased draught power_ days of reduced productivity during crop production season

1. Was the affected animal a source of income for your household? If yes, what did you sell from the affected animal?

- No
- Selling the affected animal alive
- Milk __ average milk milked per day
- Meat
- Eggs __average number of eggs per week
- Skin
- Horns
- Manure
- Renting animal power (e.g. draught power)
- Other __

1. In/for your household, was the affected animal also source of:

- Food,

If yes, what type of food:

- - Milk
  - Meat
  - Eggs
  - Blood
  - Other
- Protection
- Companion
- Draught power
- Transport of people or goods
- Direct trade
- Gifts
- Other

1. (Optional) How has your daily life been affected by the loss of your animal?

- _____________________ (open question if time remaining : answer with key words)
